# Storage and redistribution of anthropogenic CO_2_ in the western North Pacific: The role of subtropical mode water transportation

**DOI:** 10.1016/j.fmre.2022.05.001

**Published:** 2022-05-13

**Authors:** Cheng-long Li, Lei Han, Wei-dong Zhai, Di Qi, Xu-chen Wang, Hong-mei Lin, Li-wen Zheng

**Affiliations:** a Institute of Marine Science and Technology, Shandong University, Qingdao 266237, China; b China-ASEAN College of Marine Science and Technology, Xiamen University Malaysia, Selangor, Malaysia; c Southern Marine Science and Engineering Guangdong Laboratory (Zhuhai), Zhuhai 519080, China; d Polar and Marine Research Institute, Jimei University, Xiamen 361021, China; e Key Laboratory of Marine Chemistry Theory and Technology, and Frontiers Science Center for Deep Ocean Multispheres and Earth System, Ministry of Education, Ocean University of China, Qingdao 266100, China; f Center for Isotope Geochemistry and Geochronology, Pilot National Laboratory for Marine Science and Technology (Qingdao), Qingdao 266237, China; g Weihai Institute of Marine Ecology and Economy Research, Weihai 264400, China

**Keywords:** Oceanic CO_2_ uptake, Anthropogenic CO_2_ storage, Subtropical mode water, Kuroshio extension, Kuroshio recirculation, Western North Pacific

## Abstract

Oceanic uptake and storage of anthropogenic CO_2_ (C_ANT_) are regulated by ocean circulation and ventilation. To decipher the storage and redistribution of C_ANT_ in the western North Pacific, where a major C_ANT_ sink develops, we investigated the water column carbonate system, dissolved inorganic radiocarbon and ancillary parameters in May and August 2018, spanning the Kuroshio Extension (KE, 35–39 °N), Kuroshio Recirculation (KR, 27–35 °N) and subtropical (21–27 °N) zones. Water column C_ANT_ inventories were estimated to be 40.5 ± 1.1 mol m^−2^ in the KR zone and 37.2 ± 0.9 mol m^−2^ in the subtropical zone. In comparison with historical data obtained in 2005, relatively high rates of increase of the C_ANT_ inventory of 1.05 ± 0.20 and 1.03 ± 0.12 mol m^−2^ yr^−1^ in the recent decade were obtained in the KR and subtropical zones, respectively. Our water-mass-based analyses suggest that formation and transport of subtropical mode water dominate the deep penetration, storage, and redistribution of C_ANT_ in those two regions. In the KE zone, however, both the water column C_ANT_ inventory and the decadal C_ANT_ accumulation rate were small and uncertain owing to the dynamic hydrology, where the naturally uplifting isopycnal surfaces make C_ANT_ penetration relatively shallow. The findings of this study improve the understanding of the spatiotemporal variations of C_ANT_ distribution, storage, and transport in the western North Pacific.

## Highlights

• Anthropogenic CO_2_ (C_ANT_) storage was investigated in the western North Pacific.
• C_ANT_ inventories in water column and in specific water masses were estimated.
• Subtropical mode water dominates C_ANT_ dynamics in the Kuroshio Recirculation region.

## Graphical abstract

**Figure:**
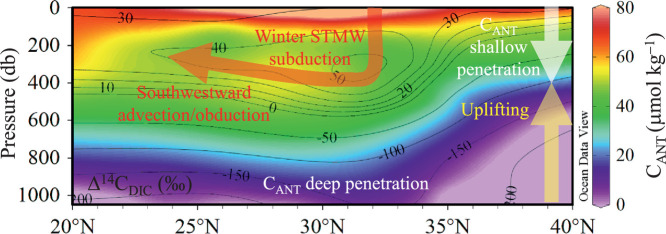

## 1 Introduction

Annually, the ocean absorbs 23%–35% of anthropogenic CO_2_ (C_ANT_) [1], [2], [3], mitigating the rise of atmospheric CO_2_ and global warming. The C_ANT_ absorbed in a specific region might be moved by the oceanic circulation and ultimately stored in another region [4], [5], [6]. Therefore, the global distribution pattern of C_ANT_ storage shows regional variations [7,8], which is different to the pattern of annual net oceanic CO_2_ uptake [9].

**Fig. 1:**
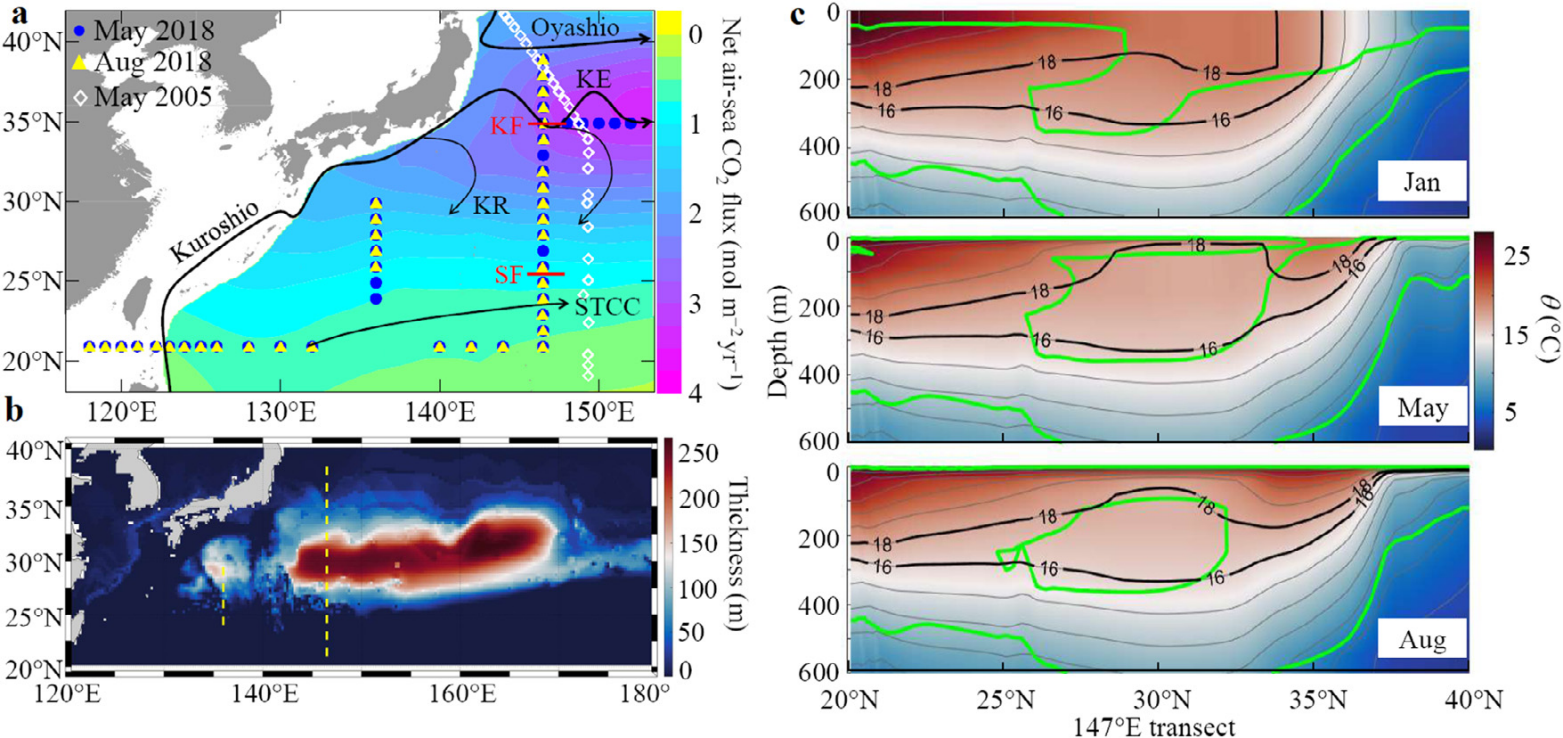
**Area maps and monthly evolution of the Subtropical Mode Water**. (a) The western North Pacific and sampling sites in May (blue circles) and August (yellow triangles) 2018, and in May 2005 (white diamonds, the P10 transect along ∼149 °E). The climatological mean annual net air–sea CO_2_ flux (mol m^−2^ yr^−1^) [9] was plotted, with positive values indicating oceanic CO_2_ uptake. Approximate locations of the Oyashio, Kuroshio, Kuroshio Extension (KE), Kuroshio Recirculation (KR), Subtropical Countercurrent (STCC), Kuroshio Front (KF) and Subtropical Front (SF) are also shown [22]. (b) The thickness of the Subtropical Mode Water defined by the criterion of density and potential vorticity (PV) [24]. (c) Potential temperature (*θ*, black contours) and PV contours of 2 × 10^−10^ m^−1^ s^−1^ (green contours) along the 147 °E transect in the North Pacific produced by the MASNUM ocean circulation model (See Supplementary materials for details).

The western North Pacific is an important and highly dynamic region (Fig. 1a), where the Kuroshio and Oyashio currents mix in the interfrontal zone off the east coast of Japan. The Kuroshio Extension (KE) plays an important role in the global ocean circulation [10,11], and also serves as the strongest sink of atmospheric CO_2_ in the North Pacific [9]. To the south of the KE, the Kuroshio Recirculation (KR) zone has the largest store of C_ANT_ in the North Pacific [7,8].

The predominant subduction process in this area is the formation of Subtropical Mode Water (STMW). It is formed in the KR zone owing to strong ventilation in winter and is then advected southwestward after subduction [12], [13], [14]. This process plays an important role both in climate variability and in C_ANT_ uptake, transportation, and storage [4,[15], [16], [17]]. However, to the best knowledge of the authors, there has been no previous data-based research quantifying the contribution of STMW to the total water column C_ANT_ inventory in the North Pacific.

Several studies have shown that the invasion of C_ANT_ into the ocean might also cause accelerated accumulation of C_ANT_ because atmospheric CO_2_ concentration has grown rapidly over past decades [18,19]. Variations of the water column C_ANT_ inventory in the recent decade are unclear and subject to the spatiotemporal resolution of the monitoring of carbonate system parameters in the western North Pacific; however, its oceanic sink for C_ANT_ over two periods (1800−1994 and 1994−2007) has been broadly quantified using field datasets along the P10 section (149 °E) [7,8]. In this study, duplicate investigations of the seawater carbonate system and its related ancillary parameters were conducted in the western North Pacific (21−39 °N, 122−152 °E) in May−June and August−September 2018. The objectives were to elucidate the spatiotemporal variations in C_ANT_ distribution and inventory, and to explore the role of STMW in deciphering the storage and redistribution of C_ANT_ in the western North Pacific. Our new dataset of carbonate system parameters might help increase the spatiotemporal resolution of oceanic C_ANT_ inventory in this region.

## 2 Materials and methods

### 2.1 Study area

In the western North Pacific, the KE is formed as the northeastward-flowing Kuroshio Current turns eastward off the coast of Japan at approximately 35 °N, 140 °E [10,11]. The KR is located to the south of the KE (Fig. 1a), where a deepened ocean mixed layer is associated with heat loss to the atmosphere [20]. To the south of the KR, the Subtropical Countercurrent (STCC) flows eastward in the shallow layer [21]. The Kuroshio front (KF) at ∼35 °N and the subtropical front (SF) at ∼27 °N divide our area of interest into three zones from north to south: the KE (35–39 °N), KR (27–35 °N), and subtropical (21–27 °N) zones [22]. As an entity, the study area behaves as a strong net sink of atmospheric CO_2_ [9].

Between the seasonal and permanent thermoclines (100−1000 m), the western North Pacific comprises mainly the STMW, Central Mode Water (CMW), and North Pacific Intermediate Water (NPIW). Owing to strong ventilation in winter, STMW is formed with a core potential temperature (*θ*) of 16−18 °C or a core potential density (σ_*θ*_) of 25.4 kg m^−3^ [12], [13], [14]. The STMW can be identified on the basis of different criteria such as *θ*, σ_*θ*_, potential vorticity (PV), and their vertical derivatives [23]. An example of how the different criteria help define this relatively uniform water mass is demonstrated for a snapshot in August along the 147 °E transect in the North Pacific from a simulation by a MASNUM ocean circulation model (Fig. S1). It can be seen that the derivative criteria best identify the boundary of the mode water. This water mass appears at approximately 30 °N at depths of 100−400 m, which is consistent with the position inferred from Argo data [24]. The horizontal extent of the simulated STMW is presented in Fig. 1b, showing concentrated distribution to the south of the KE. Remarkable seasonal evolution of the STMW is demonstrated in Fig. 1c. In winter, the subsurface water is well ventilated with the atmosphere through the subduction process caused by strong surface cooling. The STMW is continuously replenished until early spring. During summer, the STMW becomes isolated from the atmosphere owing to the formation of the strong seasonal thermocline above it. Its volume is then gradually eroded, primarily by southward advection of the subtropical gyre away from the source region.

Beneath the STMW, the CMW is characterized by a core σ_*θ*_ of 26.0 kg m^−3^, which is formed in the deep winter mixed layer at approximately 155 °E−160 °W between the KE and the subarctic front [25,26]. Both STMW and CMW are advected southward to subtropical and tropical zones after subduction. The NPIW, characterized by a core salinity minimum of <34.3 or *σ_θ_* of 26.8 kg m^−3^, is believed to form in the Okhotsk Sea and then to flow eastward and southward to the subtropical gyre through the Kuroshio–Oyashio interfrontal zone [11,27,28].

### 2.2 Sampling and analyses

The cruises for this study were undertaken in the western North Pacific onboard R/V *Xiangyanghong* 3 from May 10 to June 7, 2018, and onboard R/V *Tan Kah Kee* from August 16 to September 15, 2018 (Fig. 1a). Depth profiles of temperature and salinity (practical salinity scale of 1978) were acquired using calibrated conductivity-temperature-depth/pressure (CTD) probes (SBE911 plus, Sea-Bird Scientific, USA). During the two surveys, discrete water samples for dissolved oxygen (DO), dissolved inorganic carbon (DIC), total alkalinity (TA), and nutrients (nitrate, nitrite, phosphate and silicate) were collected mainly from the surface layer to 1000 m depth using a 10 L Niskin bottle, while samples to the bottom layers were collected only at six staions. In this study, we focused on the subsurface hydrochemical parameters between the bottom of the seasonal thermocline (∼100 m) and 1000 m depth.

Water samples for DO analyses were collected, fixed, and titrated onboard the vessels, following the standard operating procedure for Winkler titration [29]. Any possible nitrite interference in the DO titration was removed by adding 0.01% NaN_3_ during subsample fixation [30]. Methods for sampling and measurement of nutrients are presented in detail in the Supplementary Materials.

Following the well-established procedure [31], water samples for DIC and TA analyses were collected and stored in 250 mL borosilicate glass bottles. Before being sealed with greased (Apiezon-L) ground-glass stoppers, 1 mL of seawater was removed from each sample bottle to allow for thermal expansion, and 100 µL of saturated HgCl_2_ was mixed into the water samples to halt biological activity. Samples were then preserved at room temperature until determination. DIC was measured using an infrared CO_2_ detector-based DIC analyzer (AS-C3, Apollo SciTech Inc., United States), and TA was determined at 25  °C by Gran acidimetric titration using a semiautomated titrator (AS-ALK2, Apollo SciTech Inc., United States). Reproducibility of DIC and TA measurements was at the 0.1% level [32]. DIC and TA measurements were referred to certified reference materials from Andrew G. Dickson's Laboratory (Scripps Institute of Oceanography, United States) at precision of ± 2 µmol kg^−1^.

For some samples obtained in August 2018, radiocarbon measurement of DIC (^14^C_DIC_) was performed using accelerator mass spectrometry (AMS) at the Center for Isotope Geochemistry and Geochronology, Pilot National Laboratory for Marine Science and Technology (Qingdao), China. Before the ^14^C_DIC_ analysis, a sample of 60 mL of water was taken, and a self-established DIC extraction method [33,34] was performed with extraction efficiency of > 96%. For these samples, DIC data were collected immediately after the ^14^C_DIC_ sample was taken, so as to avoid any effect of gas exchange. Values of Δ^14^C_DIC_ were reported as the modern fraction, i.e., the activity ratio of a sample was normalized to the modern reference material [35], and the precision of the Δ^14^C_DIC_ analysis was 4–5‰ or better [35].

To explore the decadal changes of C_ANT_, data of temperature, salinity, DO, DIC, TA and nutrients in May 2005 along the P10 section (149 °E) [36] are employed to calculate previous C_ANT_. The Japan Meteorological Agency conducted P10 repeat observations that met the criteria of the Global Ocean Ship-based Hydrographic Investigations Panel (GO-SHIP), and the data were obtained from the U.S. NOAA website (https://www.nodc.noaa.gov/ocads/).

To quantify the effects of net community metabolism, apparent oxygen utilization (AOU) was calculated by subtracting the field-measured DO concentration from the air-equilibrated DO [37]. Assuming that DO was initially at equilibrium with the atmosphere, a value of AOU > 0 implied net community respiration, and AOU < 0 indicated net community production. To eliminate the effects of precipitation and evaporation on the seawater carbonate system, the salinity-normalized parameters (NTA and NDIC) were calculated using the following expressions: NTA = TA/salinity × 35 and NDIC = DIC/salinity × 35.

### 2.3 Calculation and comparison of anthropogenic CO_2_ using different methods

The C_ANT_ value in subsurface water was calculated using the TrOCA method [38], [39], [40]. Briefly, the method defines a quasi-conservative tracer, TrOCA (Tracer combining O_2_, DIC, and TA). TrOCA is a reasonable tracer of water masses where changes in TrOCA over time are independent of biology and can be attributed to C_ANT_ penetration. The C_ANT_ can be estimated from the difference between the current and preindustrial TrOCA (TrOCA °) divided by a stoichiometric coefficient. The simplicity of the TrOCA method relies on the fact that a simple formulation for TrOCA ° has been proposed based on *θ* and TA; thus, estimation of C_ANT_ using O_2_, DIC, TA, and *θ*. The C_ANT_ can be estimated using a formulation as follows [38]:

(1) C_ANT_ = [O_2_ + 1.279(DIC − 0.5TA) − exp(7.511 − 0.01087*θ* − 7.81 × 10^5^/TA^2^)]/1.279

The overall uncertainty of the approach is 3−6 μmol kg^−1^ owing to random propagation of the uncertainties in the parameters (O_2_, DIC, TA, and *θ*) and coefficients [38].

To evaluate the validation and precision of the TrOCA method for the western North Pacific, we compared it with the traditional ΔC* method [41,42] for calculating C_ANT_. See Supplementary Materials for details. Note that neither method is reliable within the upper mixed layer because the underlying hypotheses of each method are likely invalid because of intense biological activity and air−sea gas exchange [43,44]. The results showed that the C_ANT_ values derived using the two methods were consistent with each other at a deviation level of ± 3 µmol kg^−1^ (*n* = 1112) between 100 and 2000 m (Fig. S2), i.e., within the level of uncertainty of the TrOCA method (± 6 μmol kg^−1^), which provided confidence in the validation and precision of the TrOCA method for the western North Pacific.

### 2.4 Estimation of anthropogenic CO_2_ inventory

Water column C_ANT_ inventories in the upper 1000 m in May and August 2018 (along the 147 °E transect) and May 2005 (along the P10 transect) were calculated based on an assumption that the C_ANT_ concentrations in the upper 100 m were constant [44]; and then the C_ANT_ concentrations were integrated from the surface down to 1000 m. In the mid-2000s and late 2010s, the air-equilibrated sea surface fugacity of CO_2_ (*f*CO_2_^eq^) has increased from the preindustrial level of 280 µatm to ∼365 µatm (in the mid-2000s) and to ∼390 µatm (in the late 2010s). Attributable only to the rise in atmospheric CO_2_ and its invasion into the ocean surface (water temperature: 10−30  °C, salinity: 35, TA: 2300 µmol kg^−1^), the corresponding upper DIC increased by 47−60 µmol kg^−1^ in the mid-2000s and by 59−75 µmol kg^−1^ in the late 2010s. To simplify the inventory estimation, the upper C_ANT_ concentrations were fixed at 50 µmol kg^−1^ in 2005 and 65 µmol kg^−1^ in 2018. This simplified method introduces potential bias in the estimation of C_ANT_ of no more than 10 µmol kg^−1^ (i.e., 75 − 65 = 10 in 2018 or 60 − 50 = 10 in 2005) within the upper 100 m. Integrating this error over 100 m, the relatively small uncertainty in the C_ANT_ inventories was estimated to be 1 mol m^−2^.

### 2.5 Calculation of water column fugacity of CO_2_

Seawater fugacity of CO_2_ (*f*CO_2_) was calculated from DIC, TA, seawater temperature, salinity, phosphate, silicate, and pressure values using the CO2SYS.xls (Version 24) program [45], which is an updated version of the original CO2SYS.EXE program [46]. The carbonic acid dissociation constants [47], total boron/salinity [48], and dissociation constant of HSO_4_^−^ [49] were used to calculate *f*CO_2_.

## 3 Results

### 3.1 Data overview

**Fig. 2:**
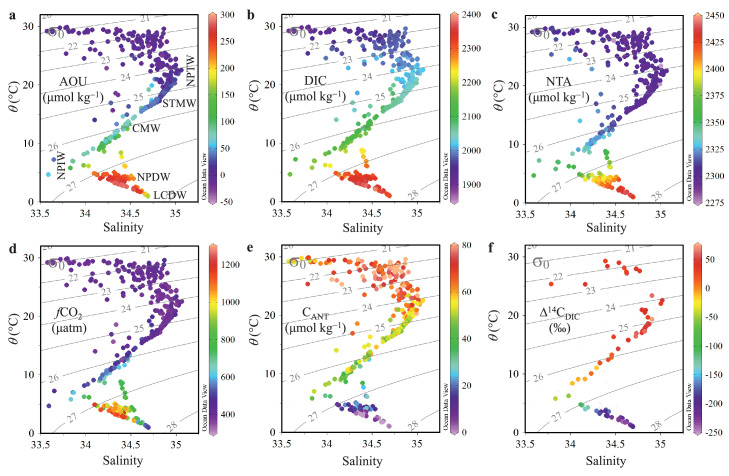
**Water column potential temperature (*****θ*****)−salinity diagram with colors indicating the apparent oxygen utilization (AOU), dissolved inorganic carbon (DIC), salinity-normalized total alkalinity (NTA), fugacity of carbon dioxide (*****f*****CO**_**2**_**), anthropogenic CO**_**2**_**(C**_**ANT**_**) and isotopic composition of radiocarbon (Δ**^**14**^**C**_**DIC**_**) in August 2018**. Gray contour lines represent the potential density referring to 0 m (σ_0_). NPTW = North Pacific Tropical Water, STMW = Subtropical Mode Water, CMW = Central Mode Water, NPIW = North Pacific Intermediate Water, NPDW = North Pacific Deep Water, LCDW = Lower Circumpolar Deep Water.

Temperature−salinity diagrams showing all data for the study area in August 2018 are presented in Fig. 2, and the hydrochemical characteristics of the subsurface water masses are listed in Table 1. The NPTW at ∼24σ*_θ_* is characterized by a subsurface salinity maximum of >34.9, low AOU/DIC/*f*CO_2_, and high TA/C_ANT_/Δ^14^C_DIC_. The NPIW with the core σ*_θ_* of 26.8 is characterized by a salinity minimum of <34.3, high AOU/DIC/TA/*f*CO_2_, and low C_ANT_/Δ^14^C_DIC_. Above the NPIW, the CMW is at the isopycnal surface of ∼26σ*_θ_*, having relatively high AOU/DIC/*f*CO_2_/C_ANT_, but the lowest TA. The STMW generally has transitional values of parameters between those of NPTW and CMW. Additionally, the STMW has nearly air-equilibrated *f*CO_2_ (402 ± 23 µatm) and considerable AOU (31 ± 9 µmol kg^−1^) in August 2018. These results imply that the STMW has lower *f*CO_2_ than the air-equilibrated value and thus it is a carbon sink in winter when the STMW is formed by strong ventilation (with an AOU of nearly zero). This conjecture is partially evidenced by our parallel observations, which showed that sea surface *f*CO_2_ in the region of STMW formation was ∼30 µatm lower than the air-equilibrated value in spring, and even ∼50 µatm lower than the air-equilibrated value in winter owing to intense seawater heat loss [32]. In the deep ocean, the NPDW and LCDW exhibit increases in salinity, TA, and Δ^14^C_DIC_ and decreases in both AOU and DIC with increasing depth.

**Table 1:** **Characteristics of water masses in the western North Pacific (east of the Luzon Strait) in August 2018**.

Waters∗	σ*_θ_*(kg m^−3^)	AOU(µmol kg^−1^)	DIC(µmol kg^−1^)	TA(µmol kg^−1^)	NTA(µmol kg^−1^)	*f*CO_2_(µatm)	TrOCA*_C*_ANT_(µmol kg^−1^)	ΔC*_C_ANT_(µmol kg^−1^)	Δ^14^C_DIC_(‰)
NPTW	24.3 ± 0.5	12 ± 16	2022 ± 18	2295 ± 7	2298 ± 7	391 ± 23	60 ± 12	64 ± 12	51 ± 18
STMW	25.2 ± 0.2	31 ± 9	2051 ± 13	2283 ± 6	2299 ± 6	402 ± 23	50 ± 5	50 ± 5	42 ± 11
CMW	26.1 ± 0.2	82 ± 21	2125 ± 24	2275 ± 6	2321 ± 8	516 ± 59	48 ± 7	47 ± 7	15 ± 18
NPIW	26.9 ± 0.3	196 ± 74	2263 ± 76	2315 ± 34	2372 ± 27	883 ± 244	26 ± 16	26 ± 16	-71 ± 55
NPDW	27.7 ± 0.1	235 ± 28	2338 ± 22	2404 ± 9	2432 ± 6	701 ± 157	-1 ± 5	-2 ± 6	-225 ± 11
LCDW	27.8 ± 0.0	176 ± 9	2301 ± 15	2409 ± 8	2430 ± 8	470 ± 39	-1 ± 3	-3 ± 3	-208 ± 10

∗NPTW = North Pacific Tropical Water, STMW = Subtropical Mode Water, CMW = Central Mode Water, NPIW = North Pacific Intermediate Water, NPDW = North Pacific Deep Water, LCDW = Lower Circumpolar Deep Water.

### 3.2 Carbonate properties and anthropogenic CO_2_

**Fig. 3:**
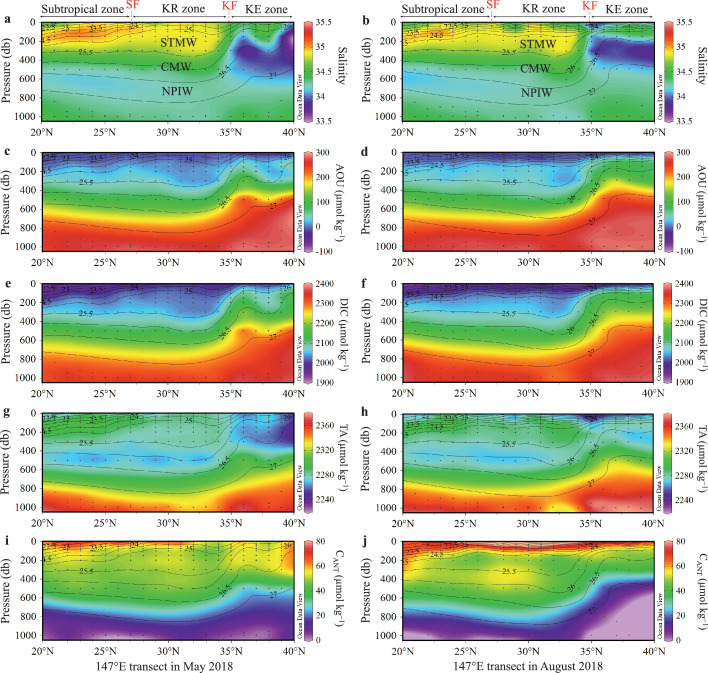
**Latitudinal variations in salinity, apparent oxygen utilization (AOU), dissolved inorganic carbon (DIC), total alkalinity (TA), and anthropogenic CO**_**2**_**(C**_**ANT**_**) along the 147 °E transect in May (left column) and August (right column) 2018**. Black contours represent the potential density. KF = Kuroshio front, SF = subtropical front, KE = Kuroshio Extension, KR = Kuroshio Recirculation, STMW = Subtropical Mode Water, CMW = Central Mode Water, NPIW = North Pacific Intermediate Water.

**Fig. 4:**
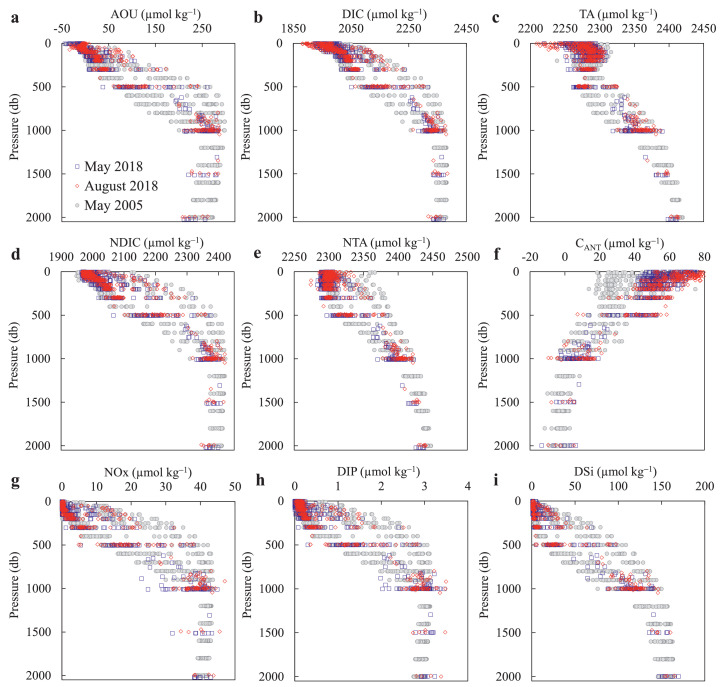
**Vertical profiles of seawater carbonate system and related parameters in May (blue rectangles) and August (red diamonds) 2018, and along the P10 transect (20**–**40** °**N, 149** °**E) in May 2005 (gray circles)**.

The latitudinal distributions of salinity, AOU, DIC, TA, and C_ANT_ between 0 and 1000 dbar along the 147 °E transect in May and August 2018 are shown in Fig. 3, and all vertical profiles of seawater carbonate system and related parameters are presented in Fig. 4.

In the KR zone, the STMW with σ_*θ*_ of 25.0−25.6 accounts for most of the water in the range of 100−400 dbar (Fig. 1c and 3a,b). In contrast, the KE zone is characterized by a vertical salinity minimum of ∼33.5 associated with the low-salinity Oyashio water mixing with the relatively saline Kuroshio water (Fig. 3a,b). Vertically, DIC increases with *σ_θ_* between 0 and 1000 dbar, but the vertical gradients of DIC and *σ_θ_* in the upper 500 dbar are weaker in the KR zone (27–35 °N) than in the KE zone (35–40 °N), indicating the effects of nearly uniform STMW. The distributions of both AOU and DIC generally exhibit consistent increase with increasing depth, indicating the effects of biological uptake in the surface waters and remineralization in deeper waters. Beneath 1500 dbar, however, AOU and DIC gradually decrease to 176 ± 9 and 2301 ± 15 µmol kg^−1^, respectively, in the LCDW (below 4000 dbar). This is because the younger LCDW (Δ^14^C_DIC_: −208‰ ± 10‰) has not accumulated as much carbon as the older NPDW (Δ^14^C_DIC_: −225‰ ± 11‰) (Table 1).

Vertical variation in NTA is different from that of DIC. In the upper 300 dbar, NTA values are nearly uniform with an average of 2299 ± 7 μmol kg^−1^ (Table 1; Fig. 4e), which is in agreement with previously reported mean NTA values of 2300 ± 6 μmol kg^−1^ [50] and 2297 ± 5 μmol kg^−1^ [18] for the western North Pacific. To further assess the quality of our data, we compared the data (along 147 °E) acquired in May and August 2018 with the earlier GO-SHIP data acquired along the P10 transect (149 °E) in May 2005. The result shows that they were consistent with each other in the deep layers (below 1000 dbar) and thus the data were considered reliable (Fig. 4 and Table S1). Therefore, no systematic adjustment was needed when we investigated the temporal variations of the carbonate system parameters between the two periods.

Seasonal variations in hydrochemical parameters below 100 dbar were mainly observed in the KE zone from May to August (Fig. 3). The isopycnal surfaces shallowed, and the subsurface water with high AOU and DIC were mixed upward and affected the seasonal variations in upper AOU and DIC.

The C_ANT_ concentration decreases from 50−70 µmol kg^−1^ at 100 dbar to approximately 0–15 µmol kg^−1^ at 1000 dbar (Fig. 3i,j, and 4f). Note that the C_ANT_ concentrations within the upper 100 dbar cannot be estimated convincingly using Eq. 1 because the intense biological activity and air–sea gas exchange likely destroy the underlying hypotheses. However, it is expected that the upper C_ANT_ concentrations are vertically homogeneous in the winter mixed layer, where C_ANT_ concentrations vary in the range of 55–75 µmol kg^−1^ (Fig. 3i,j and 4f), which is in reasonable agreement with an approximate estimate of the atmospheric-CO_2_-rise-induced surface DIC increase of 59−75 µmol kg^−1^ since the preindustrial era (Section 2.4). Along the 147 °E transect, a featured phenomenon is the deep penetration of the high C_ANT_ concentration of 40 µmol kg^−1^ at approximately 600 dbar and thus the large accumulation of C_ANT_ centered at 100−600 dbar in the KR zone.

### 3.3 Water column inventory of anthropogenic CO_2_

**Fig. 5:**
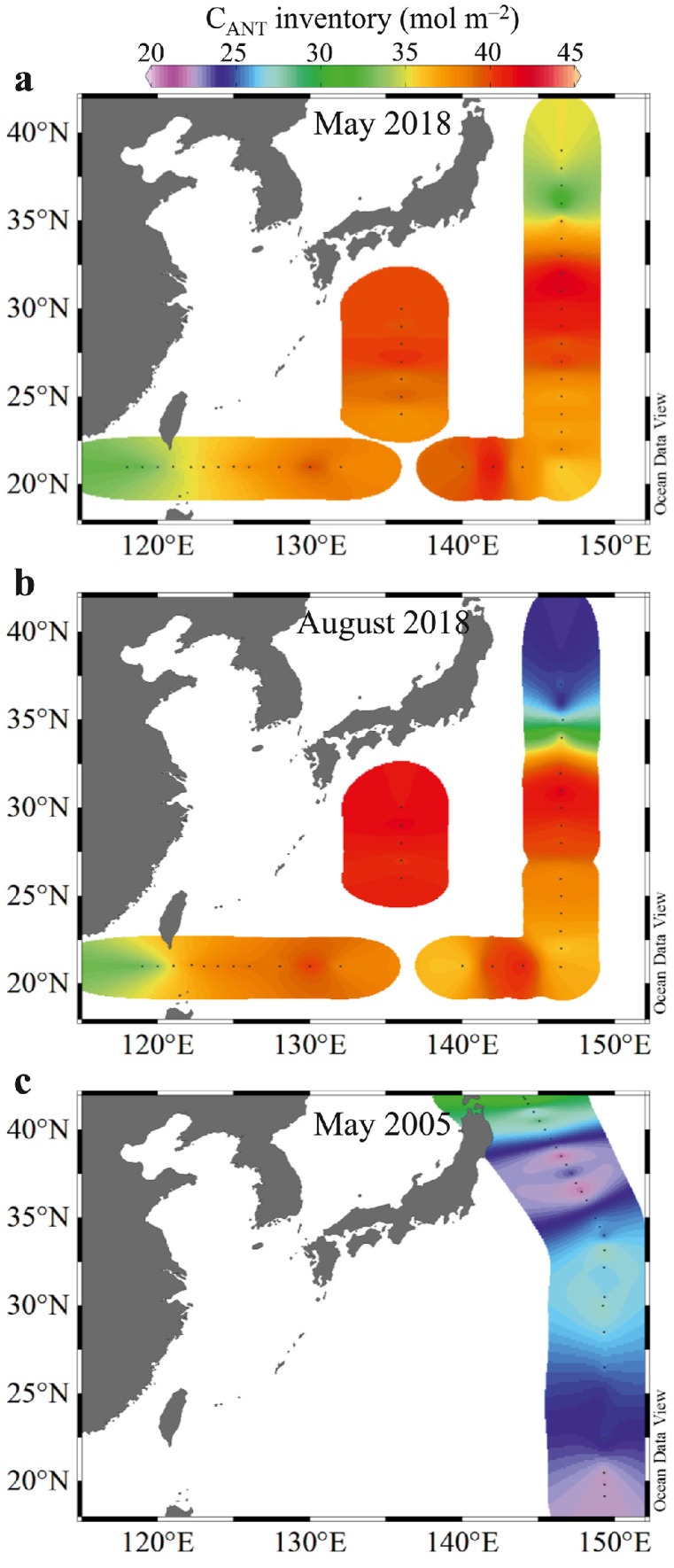
**Inventories of anthropogenic CO**_**2**_**(C**_**ANT**_**) in the western North Pacific in May and August 2018, and in May 2005**.

The water column C_ANT_ inventories in the upper 1000 dbar in May and August 2018, and May 2005 are presented in Fig. 5 and Table 2.

**Table 2:** **Water column inventories of C**_**ANT**_**along the 147 °E transect in May and August 2018 and along the 149 °E transect in May 2005**. KE = Kuroshio Extension, KR = Kuroshio Recirculation.

Zone	Inventory of C_ANT_ in May 2018 (mol m^−2^)	Inventory of C_ANT_ in August 2018 (mol m^−2^)	Inventory of C_ANT_ in May 2005 (mol m^−2^)
KE	34.0 ± 4.0	24.2 ± 1.8	23.4 ± 2.1
KR	40.4 ± 2.0	40.5 ± 1.1	26.7 ± 2.3
Subtropical	37.1 ± 0.9	37.2 ± 0.9	23.8 ± 2.0

In May 2018, the maximum C_ANT_ inventory along the 147 °E transect was 40.4 ± 2.0 mol m^−2^ in the KR zone, while a relatively low C_ANT_ inventory of 37.1 ± 0.9 mol m^−2^ was revealed in the subtropical zone; the minimum value of 34.0 ± 4.0 mol m^−2^ was found in the KE zone. In both August 2018 and May 2005, latitudinal variations in C_ANT_ inventories were similar to those in May 2018, with maxima of 40.5 ± 1.1 mol m^−2^ (in August 2018) and 26.7 ± 2.3 mol m^−2^ (in May 2005) in the KR zone, relatively low inventories of 37.2 ± 0.9 mol m^−2^ (in August 2018) and 23.8 ± 2.0 mol m^−2^ (in May 2005) in the subtropical zone, and minima of 24.2 ± 1.8 mol m^−2^ (in August 2018) and 23.4 ± 2.1 mol m^−2^ (in May 2005) in the KE zone. These results reflect a similar distribution pattern to that of the long-term C_ANT_ accumulation in the western North Pacific [7,8].

## 4 Discussion

### 4.1 Anthropogenic CO_2_ inventory in specific water masses along the 147 °E transect

**Fig. 6:**
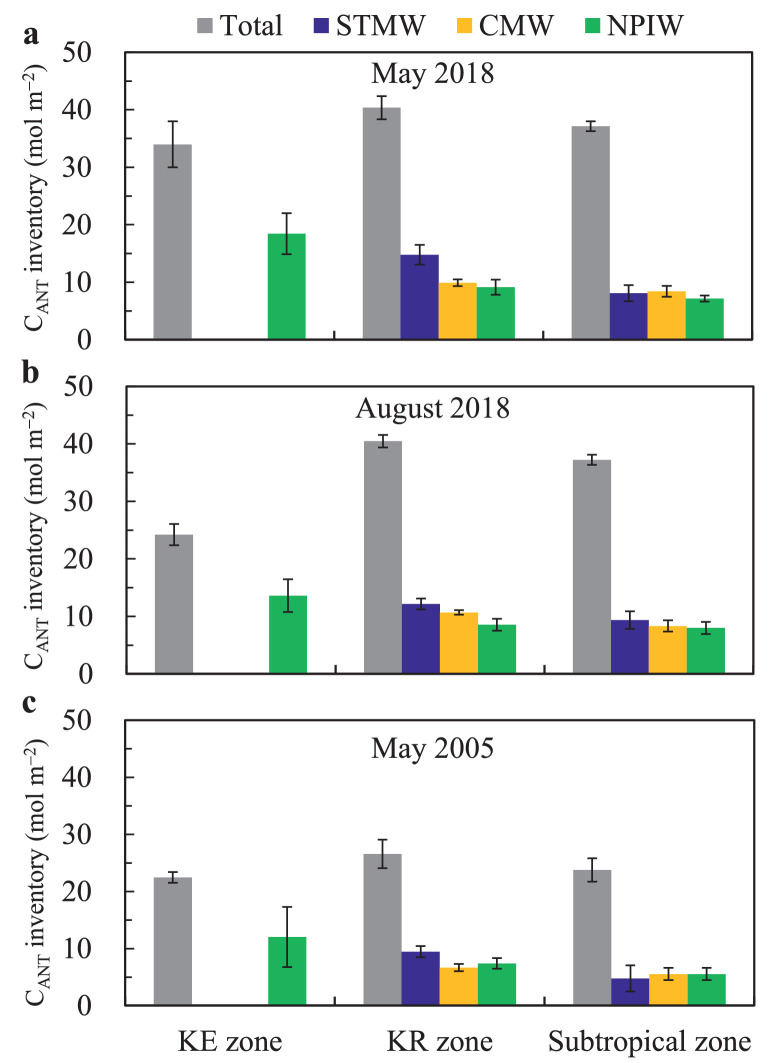
**Inventories of anthropogenic CO**_**2**_**(C**_**ANT**_**) in specific water masses delimited by the σ***_**θ**_***intervals: the Subtropical Mode Water (STMW) within 25σ***_**θ**_***−25.6σ***_**θ**_***, Central Mode Water (CMW) within 25.7σ***_**θ**_***−26.4σ**_***θ***_**, and North Pacific Intermediate Water (NPIW) within 26.4σ***_**θ**_***−27.2σ***_**θ**_***in the Kuroshio Extension (KE, 35–39 °N), Kuroshio Recirculation (KR, 27–35 °N), and subtropical (21–27 °N) zones along 147 °E in May and August 2018 and along the P10 (149 °E) transect in May 2005**. Error bars denote standard deviations.

To quantify the contributions of C_ANT_ inventories in the water masses to the water column C_ANT_ inventories in the western North Pacific, we calculated specific C_ANT_ inventories for the three water masses on the basis of σ_*θ*_ intervals: STMW within the σ_*θ*_ range of 25−25.6 kg m^−3^, CMW within the σ_*θ*_ range of 25.7−26.4 kg m^−3^, and NPIW within the σ_*θ*_ range of 26.4−27.2 kg m^−3^. The latitudinal variations of specific C_ANT_ inventories in the three water masses and their contributions to the water column C_ANT_ inventories are presented in Table 3 and Fig. 6.

**Table 3:** **Inventories of anthropogenic CO**_**2**_**(C**_**ANT**_**) in specific water masses in the KE (35–39 °N), KR (27–35 °N), and subtropical (21–27 °N) zones**. KE = Kuroshio Extension, KR = Kuroshio Recirculation, STMW = Subtropical Mode Water, CMW = Central Mode Water, NPIW = North Pacific Intermediate Water.

Survey	Zone	C_ANT_ inventory (mol m^−2^)			Proportion (%)		
		STMW	CMW	NPIW	STMW	CMW	NPIW
May 2018	KE	/	/	18.4±3.6	/	/	55%±10%
	KR	14.8 ± 1.7	9.9 ± 0.6	9.1 ± 1.3	37% ± 5%	25% ± 2%	23% ± 2%
	Subtropical	8.1 ± 1.4	8.4 ± 1.0	7.2 ± ± 0.5	22% ± 2%	23% ± 2%	19% ± 1%
August 2018	KE	/	/	13.6 ± 2.9	/	/	56% ± 10%
	KR	12.2 ± 1.0	10.7 ± 0.4	8.5 ± 1.0	30% ± 2%	26% ± 1%	21% ± 2%
	Subtropical	9.4 ± 1.5	8.3 ± 1.0	8.0 ± 1.1	25% ± 4%	22% ± 2%	21% ± 3%
May 2005	KE	/	/	13.9 ± 6.0	/	/	59% ± 23%
	KR	9.1 ± 1.3	6.7 ± 0.6	7.4 ± 0.8	34% ± ± 4%	25% ± 3%	28% ± 2%
	Subtropical	4.8 ± 2.3	5.5 ± 1.1	5.5 ± 1.1	20% ± 8%	23% ± 4%	23% ± 3%

Along the 147 °E transect in May 2018, the C_ANT_ inventory of STMW decreased from 14.8 ± 1.7 mol m^−2^ in the KR zone to 8.1 ± 1.4 mol m^−2^ in the subtropical zone owing to the thicker mode water in its major formation region (KR zone) than that in the subtropical zone (Fig. 3 and Table 3), whereas the C_ANT_ inventories of CMW and NPIW had minor variations in the KR and subtropical zones owing to the relatively unchanged thickness of the relevant waters. Correspondingly, the C_ANT_ inventory of STMW accounted for 37% ± 5% and 22% ± 2% of the water column C_ANT_ inventory in the KR and subtropical zones, respectively, whereas the CMW and NPIW made comparable contributions (∼22%) to the water column C_ANT_ inventories in the KR and subtropical zones. Vertically, the C_ANT_ concentration of both STMW and CMW was apparently higher than that of NPIW. However, the NPIW (26.4−27.2σ_*θ*_) represented approximately half the total volume in the upper 1000 m of the North Pacific subtropical gyre [11,27,28], leading to a large C_ANT_ pool even though the C_ANT_ concentration was relatively low [7,51].

In August 2018 along the 147 °E transect and in May 2005 along the P10 transect (149 °E), the latitudinal variations of specific C_ANT_ inventories in the STMW, CMW, and NPIW, and their contributions to the water column C_ANT_ inventories, were generally similar to those in May 2018 (Fig. 3 and Table 3). Overall, the STMW was the largest contributor to the formation of water column C_ANT_ inventory in the KR zone, whereas in the subtropical zone, STMW, CMW, and NPIW made comparable contributions to water column C_ANT_ inventory.

The volume of STMW showed seasonal variation [23,24], indicating that the C_ANT_ inventory of STMW was also subject to seasonal variation. From May to August 2018, the C_ANT_ inventory of STMW decreased by ∼2.6 mol m^−2^ owing to the decline of its thickness in the KR zone (Fig. 3 and Table 3). However, on a long-term scale, STMW is formed in the KR zone every winter [12], [13], [14]; moreover, the AOU-corrected and salinity-normalized DIC of STMW has generally followed the track of atmospheric CO_2_ increase over the previous 20 years [15]. These results indicate that the C_ANT_ inventory of STMW has increasd continually over the past several decades, and also support the idea that mode water formation is a key process in the natural sequestration of C_ANT_ [4,17,52].

There is no mode water in the KE zone, and the C_ANT_ inventory of NPIW accounted for approximately 57% of the water column C_ANT_ inventory in the three cruises (Table 3). From May to August 2018, the water column C_ANT_ inventories decreased substantially by ∼10 mol m^−2^, and the C_ANT_ inventory of NPIW decreased by ∼5 mol m^−2^, indicating the effects of physical processes such as ocean circulation and/or movement of mesoscale eddies. Indeed, the KE zone is a highly dynamic region with intense mesoscale eddies [11,53,54], which might affect the C_ANT_ inventory in the region. Sea surface height data showed that a warm mesoscale eddy moved into the region of the 147 °E transect of the KE zone in May 2018, and disappeared in August 2018 (Fig. S3). Correspondingly, the isolines of water hydrochemical parameters and C_ANT_ in the upper layer between 35 °N and 40 °N were notably depressed in May in comparison with those in August of 2018 (Fig. 3). This remarkable deepening signature is consistent with the baroclinic structure of a warm eddy, and its horizontal scale agrees with that of a warm eddy detected in May by altimetry data. Therefore, the difference between May and August in the C_ANT_ inventories in the water column and NPIW might have been induced by the perturbation of the mesoscale eddy.

### 4.2 Decadal variations of anthropogenic CO_2_ inventory along the 147 °E transect

Temporal variations of water column and specific C_ANT_ inventories between the 147 °E and P10 (149 °E) transects from May 2005 to May/August 2018 were calculated based on Tables 2 and 3. In the dynamic KE zone, the decadal variations of C_ANT_ inventories were uncertain because of the eddy-induced perturbation (Section 4.1). In this section, we mainly discuss the decadal variations of the C_ANT_ inventory in the main formation region of STMW (i.e., the KR zone). Our results show that the rate of increase of water column C_ANT_ inventories in the KR zone (27–35 °N, 147–149 °E) was 1.05 ± 0.20 mol m^−2^ yr^−1^ between May 2005 and May 2018, or 1.07 ± 0.20 mol m^−2^ yr^−1^ between May 2005 and August 2018. This rate is very close to that observed in the subtropical South Pacific Ocean and the subtropical South Indian Ocean between the 1990s and the 2000s (∼1.0 mol m^−2^ yr^−1^) [55,56], but is approximately 0.7 times greater than the rate detected in the nearby region (24–30 °N, 149 °E) and the subtropical South Atlantic Ocean between the 1990s and the 2000s (i.e., ∼0.60 mol m^−2^ yr^−1^) [57,58]. The rate of increase of the C_ANT_ inventory to the south of the KE between 1994 and 2007 was also estimated to be 0.6−0.7 mol m^−2^ yr^−1^ [7]. Overall, in the KR zone, the increase of the C_ANT_ inventory between 2005 and 2018 was 75% faster than that between the 1990s and the 2000s.

In the KR zone, the rate of increase of the C_ANT_ inventory in the STMW, CMW, and NPIW was 0.44 ± 0.14, 0.36 ± 0.06, and 0.19 ± 0.19 mol m^−2^ yr^−1^, respectively, accounting for 43% ± 16%, 35% ± 8%, and 17% ± 17% of the total rate of increase of the water column C_ANT_ inventory (1.05 ± 0.20 mol m^−2^ yr^−1^), respectively. Moreover, between May 2005 and May 2018, the thickness of STMW had minor variations, whereas the C_ANT_ concentrations of STMW substantially increased from 27 ± 3 to 48 ± 3 μmol kg^−1^ at a rate of 1.62 ± 0.16 μmol kg^−1^ yr^−1^. This rate is consistent with the rate of increase of surface NDIC during 2008–2017 (1.68 ± 0.23 μmol kg^−1^ yr^−1^) in the western part of the KR zone (26–30 °N, 137 °E) [18], but is approximately 0.7 times greater than the rate of increase of C_ANT_ concentration of STMW in the nearby region (24–30 °N, 149 °E) between 1993 and 2005 (0.97 ± 0.10 µmol kg^−1^ yr^−1^ [57]), which is consistent with the value expected from consideration of the rate of atmospheric CO_2_ increase (0.95 µmol kg^−1^ yr^−1^). These results indicate that the acceleration in the rate of increase of water column C_ANT_ inventory in the KR zone can be attributed mainly to the C_ANT_ increase of the STMW.

To explore the C_ANT_ increase of the STMW, we modeled the increase of C_ANT_ considering only the atmospheric CO_2_ increase between 2005 and 2018, with an increase of atmospheric CO_2_ mole fraction (*x*CO_2_) from 380 ×10^−6^ to 409 × 10^−6^ (sample collected at Mauna Loa, http://www.esrl.noaa.gov/gmd/), and at a water temperature of 18 °C, salinity of 34.8, and TA of 2285 μmol kg^−1^. The modeled result shows that the air-equilibrated increase rate of C_ANT_ was 1.07 μmol kg^−1^ yr^−1^, which accounted for 66% of the observed rate of increase of 1.62 ± 0.16 μmol kg^−1^ yr^−1^ in the STMW.

Globally, the variability of oceanic CO_2_ uptake over the past three decades was likely attributable to the variability of the upper-ocean overturning circulation [59], which drives the air−sea CO_2_ gradient and thereby oceanic CO_2_ uptake. For the entire Pacific, only ∼50% of the acceleration of the C_ANT_ storage during 2005–2015 could be attributed to the increase in atmospheric CO_2_ [60]. In the Southern Pacific, the recent acceleration of the C_ANT_ was likely because of enhanced ventilation of the Southern Pacific Subtropical Cell [52]. In summary, C_ANT_ accumulation in the ocean interior between the 2000s and the 2010s has exceeded expectations based on the rise of atmospheric CO_2_. We provide a new case study in the KR zone to support this argument. However, the roles of ocean circulation and ventilation in regulating C_ANT_ variability require further study.

### 4.3 Redistribution of anthropogenic CO_2_ via subtropical mode water transportation

**Fig. 7:**
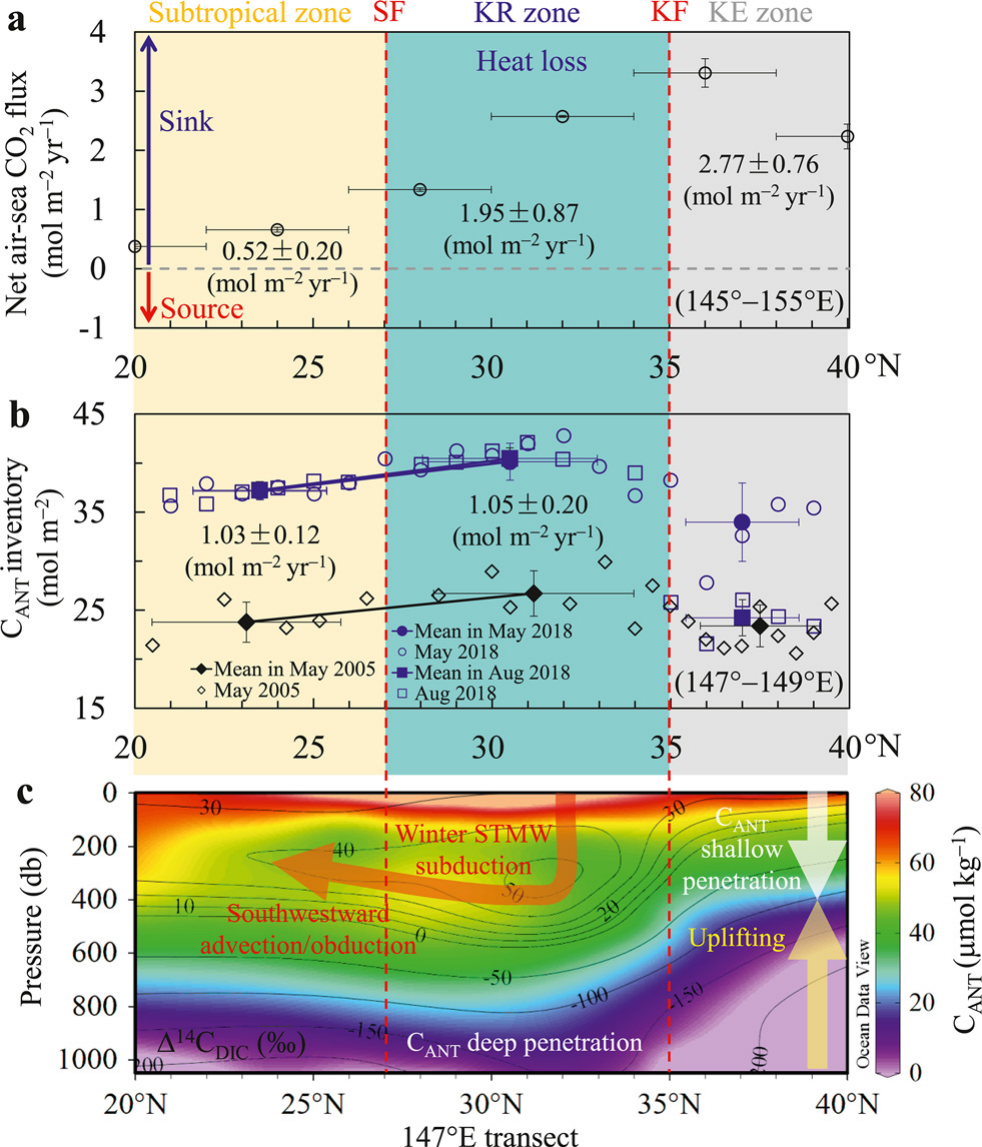
**Meridional distributions of air–sea CO**_**2**_**flux, water column inventories of anthropogenic CO**_**2**_**(C**_**ANT**_**) and the isotopic composition of radiocarbon crossing the Kuroshio Extension (KE), Kuroshio Recirculation (KR) and subtropical zones**. (a) Latitudinal variations in climatological mean annual net air–sea CO_2_ flux (mol m^−2^ yr^−1^) along 145–155 °E based on literature data [9]. Positive values indicate that the ocean serves as a sink of atmospheric CO_2_. (b) Latitudinal variations in water column inventories of C_ANT_ along the 147–149 °E transects in May and August 2018 and in May 2005. (c) Sketch of the latitudinal C_ANT_ redistribution (colors) together with the Subtropical Mode Water transport as traced by the isotopic composition of radiocarbon (Δ^14^C_DIC_, collected along the 147 °E transect in August 2018) (isolines) in the western North Pacific. KF = Kuroshio front, SF = subtropical front.

The global pattern of the C_ANT_ inventory is different to that of the air−sea CO_2_ flux [5,61]. The discrepancy between the patterns of uptake and accumulation is largely attributable to transport of C_ANT_ within the ocean interior from high-latitude regions where C_ANT_ is primarily taken up from the atmosphere to the mid-latitude thermoclines where it is primarily stored [6], [7], [8]. In the western North Pacific along the 147 °E/149 °E transect, the air−sea CO_2_ flux in the KE zone (2.77 ± 0.76 mol m^−2^ yr^−1^) was found to be larger than that in either the KR zone (1.95 ± 0.87 mol m^−2^ yr^−1^) or the subtropical zone (0.52 ± 0.20 mol m^−2^ yr^−1^) (Fig. 1a and 7a). However, the C_ANT_ inventory in the KE zone was lower than that in the KR and subtropical zones during the three surveys in May 2005, and May and August 2018 (Fig. 7b).

To reveal the controlling mechanisms of C_ANT_ transport in the western North Pacific, we used Δ^14^C_DIC_ to further trace the C_ANT_ dynamics. For tropical and mid-latitude waters in the Pacific Ocean, the pre-bomb (i.e., pre-1958) Δ^14^C_DIC_ value in the surface water has been determined previously to be −50‰ [62], [63], [64]. The isoline of Δ^14^C_DIC_ = −50‰ is shown in Fig. 7c. Its depth was located at approximately 600 dbar to the south of the KF, while it decreased to approximately 300 dbar to north of the KF. The waters above this isoline have been contaminated by bomb-produced ^14^C.

Although the shape of the bomb Δ^14^C_DIC_ contours is similar to that of C_ANT_ (Fig. 3i−j), the C_ANT_ has penetrated much deeper than the bomb ^14^C. This is because the C_ANT_ was produced over a century before the addition of the substantial bomb ^14^C (in the 1950s). Corresponding to the deep penetration and large C_ANT_ inventory in the KR zone, the area is characterized by STMW formation (Fig. 1c) and higher bomb Δ^14^C_DIC_ concentrations (Fig. 7c).

Moreover, to the south of the KF, the tongue-like distributions of Δ^14^C_DIC_ in the upper 400 dbar indicate C_ANT_ transport (Fig. 7c), which followed the subduction, southwestward advection/obduction, and vertical diffusion of the STMW [12], [13], [14]. The STMW is formed in winter and then mixed with the relatively high bomb Δ^14^C_DIC_ in the surrounding waters, introducing the high bomb Δ^14^C_DIC_ signals further into the ocean interior. Thus, the formation and transportation of STMW are responsible for the deep penetration of both the bomb Δ^14^C_DIC_ and the C_ANT_ in the KR zone, while the uplifting isopycnal surfaces in the KE zone cause shallow penetration of bomb Δ^14^C_DIC_ and C_ANT_. Similarly, several previous studies conducted at subtropical latitudes have found that the mode-water-formation-induced C_ANT_ penetration in the western Pacific is deeper than that in eastern Pacific regions that feature upwelling [41,[65], [66], [67]].

In the KR zone, the net annual air−sea CO_2_ flux is larger than the rate of increase of the C_ANT_ inventory by 0.88 mol m^−2^ yr^−1^ (Fig. 7a versus 7b). In the subtropical zone, however, the net annual air−sea CO_2_ flux is lower than the rate of increase of the C_ANT_ inventory by 0.51 mol m^−2^ yr^−1^ (Fig. 7a versus 7b). Interestingly, the rate of increase of the C_ANT_ inventory in the subtropical zone (1.03 ± 0.12 mol m^−2^ yr^−1^) is comparable with that in the KR zone (1.05 ± 0.20 mol m^−2^ yr^−1^). This result indicates that C_ANT_ absorbed in the KR zone moves into the subtropical zone via southward transportation of C_ANT_-rich STMW, as traced by the Δ^14^C_DIC_ data (Fig. 7c). In summary, the latitudinal gradient of the C_ANT_ inventory is mainly dominated by the coupling of STMW formation in the KR zone, STMW transportation in the subtropical zone, and uplifting isopycnal surfaces in the KE zone.

## 5 Conclusion

In the western North Pacific in August 2018, the C_ANT_ inventory exhibited a substantial latitudinal gradient, with a maximum value of 40.5 ± 1.1 mol m^−2^ in the KR zone (27−35 °N), a relatively low inventory of 37.2 ± 0.9 mol m^−2^ in the subtropical zone (21−27 °N), and a minimum value of 24.2 ± 1.8 mol m^−2^ in the KE zone (35−40 °N). In the KR zone, the STMW is the largest contributor to the formation of water column C_ANT_ inventory, and C_ANT_ transport within the STMW was revealed using dissolved inorganic radiocarbon as a tracer. The result also supports the idea that STMW formation is a key process in natural C_ANT_ sequestration. In the KR zone, the water column C_ANT_ inventory increased by 1.05 ± 0.20 mol m^−2^ yr^−1^ between 2005 and 2018, which was 75% higher than that observed between the 1990s and the 2000s (0.6 ± 0.2 mol m^−2^ yr^−1^), and also higher than that predicted solely on the basis of the rise in atmospheric CO_2_. In the dynamic KE zone, however, the decadal variations of the C_ANT_ inventories remain uncertain. This work improves our understanding of the spatiotemporal variations of C_ANT_ concentration and inventory in the western North Pacific.

## Declaration of competing interest

The authors declare that they have no conflicts of interest in this work.

## Appendix Supplementary materials

Image, application 1
