# Supplementary material for: Storage and redistribution of anthropogenic CO2 in the western North Pacific: The role of subtropical mode water transportation
Source: Fundam Res. 2022 May 13;4(1):103–12. doi: 10.1016/j.fmre.2022.05.001 (PMC11197630; doi:10.1016/j.fmre.2022.05.001)
Supplement: Supplementary file 1 [file mmc1.pdf]

Supplementary Materials for

Storage and redistribution of anthropogenic CO_2 in the western North Pacific: The role of Subtropical Mode Water transportation

Cheng-long Li[a], Lei Han[b], Wei-dong Zhai[a,c,*], Di Qi[d], Xu-chen Wang[e,f], Hong-mei Lin[a], Li-wen Zheng[g]

[a] Institute of Marine Science and Technology, Shandong University, Qingdao 266237, China
[b] China-ASEAN College of Marine Science and Technology, Xiamen University Malaysia, Selangor, Malaysia
[c] Southern Marine Science and Engineering Guangdong Laboratory (Zhuhai), Zhuhai 519080, China
[d] Polar and Marine Research Institute, Jimei University, Xiamen 361021, China
[e] Key Laboratory of Marine Chemistry Theory and Technology, and Frontiers Science Center for Deep Ocean Multispheres and Earth System, Ministry of Education, Ocean University of China, Qingdao 266100, China
[f] Center for Isotope Geochemistry and Geochronology, Qingdao National Laboratory for Marine Science and Technology, Qingdao 266237, China
[g] Weihai Institute of Marine Ecology and Economy Research, Weihai 264400, China

* Corresponding author: wdzhai@126.com (W. Zhai).

Supplementary materials include:

Introduction to Simulation of the North Pacific Subtropical Mode Water

Introduction to Sampling and analytical analyses for nutrients

Comparison of anthropogenic CO_2 estimations from different methods

Table S1. Characteristics of deep waters (~1000 m) in the western North Pacific (21°–35°N, 123°–147°E) in May and August 2018 and May 2005. Note that "(Aug 2018 – May 2005)" indicates the differences in parameters between August 2018 and May 2005.

Fig. S1. Different criteria used to define the Subtropical Mode Water along the 147°E transect in the North Pacific.
Fig. S2. Comparison of anthropogenic CO_2 values calculated using different methods.
Fig. S3. Monthly mean sea surface height (m) in March, April, May, and August 2018.

Reference

Simulation of the North Pacific Subtropical Mode Water

To examine the possible mechanism of subducted C_{ANT}, the characteristics of the North Pacific Subtropical Mode Water (STMW) were simulated using the MASNUM three-dimensional, primitive equation, numerical ocean circulation model. This model adopts most of the numerical schemes of the Princeton Ocean Model (POM) but uses a two-time-level algorithm for time stepping. This modification helps remove the computational mode inherent in the three-time-level scheme such as the leapfrog method adopted by POM and many other ocean circulation models [1]. In addition to inheriting other aspects of the numerical features of POM, MASNUM exhibited better performance in terms of numerical stability [2-4].

In this case, a MASNUM global simulation with horizontal resolution of 0.25° × 0.25° and 31 sigma levels was run under the GEBCO 08 topography (www.gebco.net). The simulation started from rest with initial temperature and salinity distributions obtained from WOA09 [5-6] and forced by surface fluxes of the NCEP-DOE AMIP-II Reanalysis [7]. The model was integrated until the total kinetic energy of the global ocean reached a quasi-equilibrium state [2]. The upper ocean where the STMW exists was fully spun up to depict the seasonal evolution of this water mass.

Sampling and analytical analyses for nutrients

Descriptions of the methods used for sampling and determination of discrete water samples for nutrients (nitrate, nitrite, phosphate, and silicate) in May 2018 are given below. In the surveys, duplicate water samples were unfiltered and stored in 60 mL high-density polyethylene bottles. The samples were frozen at −20 °C and then analyzed later in the laboratory for nitrate, nitrite, dissolved inorganic phosphate (DIP), and dissolved silicate (DSi). Nitrate (NO_3^-) and nitrite (NO_2^-) were measured by reducing nitrate to nitrite with a cadmium column, followed by determination of nitrite using the pink azo dye spectrophotometric method [8]. DIP and DSi were measured based on standard phosphomolybdenum blue (for DIP) and silicon molybdenum blue (for DSi) spectrophotometric procedures [9]. Nitrate, nitrite, DIP, and DSi were determined using a reorganized Syslyzer III analyzer (Systea S.P.A. Co., Italy). Detection limits were 0.1 μmol L^{-1} for nitrate, 0.04 μmol L^{-1} for nitrite, 0.08 μmol L^{-1} for DIP, and 0.08 μmol L^{-1} for DSi [10]. Samples with high nutrient concentrations were properly diluted before determination. For data quality assurance, Reference Materials for Nutrients in Seawater from The General Environmental

Technos Co., Ltd. (http://www.kanso.co.jp/) were used during the analyses, and a precision level of ± 0.1 µmol L^{-1} was achieved for most nutrient species other than ammonium.

Comparison of anthropogenic CO_2 estimations from different methods

To evaluate the validation and precision of the TrOCA method in relation to the North Pacific, we compared it with the traditional ΔC* method used for calculating C_{ANT} [11-12]. Briefly, the ΔC* method can be expressed as follows:

$$C_{ANT} = \Delta C^* - \Delta C_{diseq} , \quad (S1)$$

$$\Delta C^* = C_m - C_{280} - \Delta C_{bio} , \quad (S2)$$

where the quasi-conservative tracer (ΔC*) is defined as the difference between the measured DIC (C_m) corrected for biology (ΔC_{bio}) and the air-equilibrated DIC with a preindustrial atmosphere level of CO_2 of 280 µatm at the water surface (C_{280}); ΔC_{diseq} is the air–sea CO_2 difference expressed in terms of DIC, and the mean value of −6 ± 3 µmol kg^{-1} for the North Pacific [11] was used to calculate the C_{ANT}; ΔC_{bio} is the DIC change attributable to remineralization of organic matter and the dissolution of calcium carbonate particles and denitrification:

$$\Delta C_{bio} = 117/170 \times AOU + 0.5\ (TA - TA^\circ + 16/170 \times AOU) - 106/104 \times (N^* - N^*_{mean}), \quad (S3)$$

where 117/170 and 16/170 are the C/O_2 and N/O_2 ratios, respectively [13]. The denitrification term was 106/104 × ($N^* - N^*_{mean}$) [14], where N* = (N − 16P + 2.90) and the mean N* (N^*_{mean}) value for our dataset was 0 µmol kg^{-1}. In this study, TA° was estimated for the North Pacific using a multiple linear regression of the surface alkalinity values to conservative tracers:

$$TA^\circ = 148.7 + 61.36 \times Salinity + 16/170 \times (O_2 + 170 \times P) - 0.582 \times \theta, \quad (S4)$$

where 170 is the O_2/P ratio and θ is potential temperature.

The C_{ANT} values calculated using the TrOCA method (TrOCA_C_{ANT}) and the ΔC* method (ΔC*_C_{ANT}) were comparable with each other (Fig. S3). The result showed that the C_{ANT} values obtained using the two methods were consistent with each other at deviation levels of ± 3 µmol kg^{-1} (n = 1112) between 100 and 2000 m, within the uncertainty of the TrOCA method (± 6 µmol kg^{-1}), which provided confidence in the validation and precision of the TrOCA method for the western North Pacific.

Table S1. Characteristics of deep waters (~1000 m) in the western North Pacific (123°–147°E, 21°–35°N) in May and August 2018 and May 2005. Note that "(Aug 2018 – May 2005)" indicates the differences in parameters between August 2018 and May 2005.

Time /Difference	Pressure (db)	AOU (μmol kg^{-1})	DIC (μmol kg^{-1})	TA (μmol kg^{-1})	C_{ANT} (μmol kg^{-1})	NOx (μmol kg^{-1})	DIP (μmol kg^{-1})	DSi (μmol kg^{-1})	Number
Aug 2018	1007±6	257±13	2332±17	2357±12	8±8	38.7±2.9	2.88±0.20	114±11	n=33
May 2018	1008±2	256±14	2324±12	2350±13	7±7	38.9±3.9	2.93±0.28	115±12	n=38
May 2005	1000±1	256±21	2319±24	2345±12	7±5	39.9±1.9	2.89±0.13	107±10	n=12
Aug 2018 – May 2005	-1	1	13	12	1	-1.2	-0.01	7	/
May 2018 – May 2005	-2	0	6	5	0	-1.0	0.03	9	/

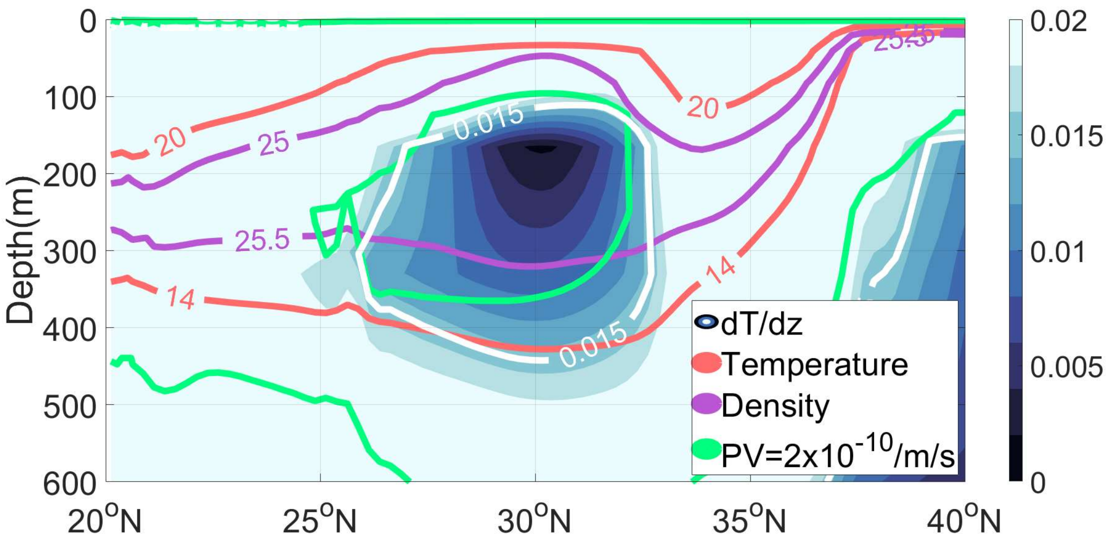

Fig. S1. Different criteria used to define the Subtropical Mode Water along the 147°E transect in the North Pacific. Color fills denote the vertical derivative of the potential temperature, dT/dz. Pink, purple, and green lines represent contour lines of potential density, temperature, and potential vorticity (PV = 2×10^{-10} m^{-1} s^{-1}), respectively. White lines denote the dT/dz criterion [15]. The data were produced by a global run of the MASNUM ocean circulation model [2].

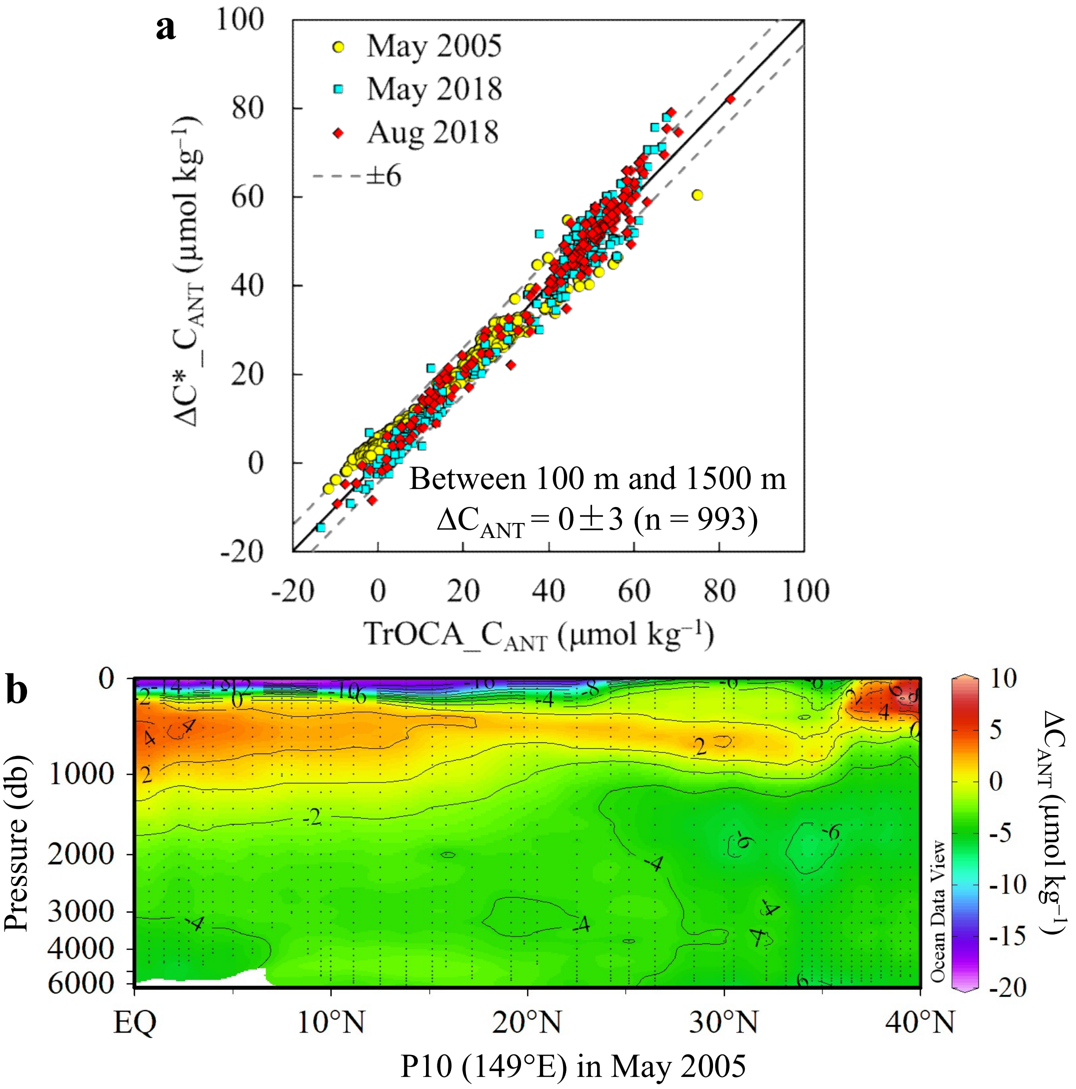

Fig. S2. Comparison of C_{ANT} values calculated using the TrOCA method (TrOCA_C_{ANT}) and ΔC* method (ΔC*_C_{ANT}) between 100 and 2000 m in May and August 2018 and in May 2005 along the P10 transect (0°–40°N, 149°E) in the western North Pacific. Here, ΔC_{ANT} (mean ± S.D.) means the TrOCA_C_{ANT} concentration minus the ΔC*_C_{ANT} concentration.

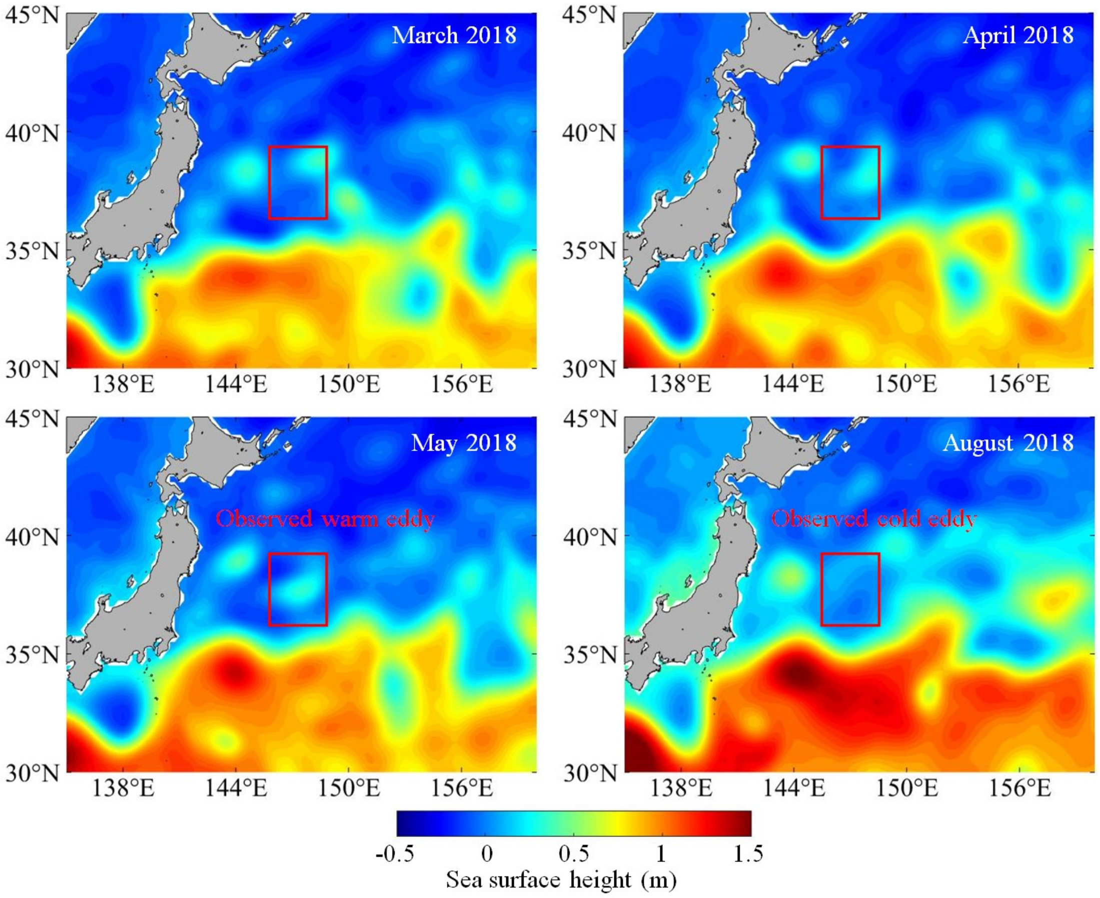

Fig. S3. Monthly mean sea surface height (m) in March, April, May, and August 2018 obtained from https://marine.copernicus.eu (red rectangles indicate the region where a mesoscale eddy was observed in May 2018).

References

[1] L. H. Kantha, C. A. Clayson. Numerical Models of Oceans and Oceanic Processes. International Geophysics Series 66 (2000) Academic Press, San Diego, USA. 750 pp. https://www.sciencedirect.com/bookseries/international-geophysics/vol/66/suppl/C

[2] L. Han. A two-time-level split-explicit ocean circulation model (MASNUM) and its validation. Acta Oceanol. Sin. 33 (11) (2014) 11–35. https://doi.org/10.1007/s13131-014-0553-z

[3] Z.-P. Zhuang, Y.-L. Yuan, J. Zhang, et al. An efficient parallel algorithm for ocean circulation numerical model based on irregular rectangle decomposition scheme. Acta Oceanol. Sin. 35 (4) (2016) 18–23. https://doi.org/10.1007/s13131-016-0855-4

[4] Z.-P. Zhuang, Y.-L. Yuan, G.-B. Yang. An ocean circulation model in σS-z-σB hybrid coordinate and its validation. Ocean Dynam. 68 (2018) 159–175. https://doi.org/10.1007/s10236-017-1124-6

[5] J. I. Antonov, J. I., D. Seidov, D., T. P. Boyer, et al. World Ocean Atlas 2009, Volume 2: Salinity. In: S. Levitus (Ed.) NOAA Atlas NESDIS 69, U.S. Government Printing Office, Washington DC, USA (2010). 184 pp. https://www.nodc.noaa.gov/OC5/indprod.html

[6] R. A. Locarnini, A. V. Mishonov, J. I. Antonov, et al. World Ocean Atlas 2009, Volume 1: Temperature. In: S. Levitus (Ed.) NOAA Atlas NESDIS 68, U.S. Government Printing Office, Washington DC, USA (2010). 184 pp. https://www.nodc.noaa.gov/OC5/indprod.html

[7] M. Kanamitsu, W. Ebisuzaki, J. Woollen, et al. NCEP–DOE AMIP-II Reanalysis (R-2). B. Am. Meteorol. Soc. 83 (11) (2002) 1631−1644. https://doi.org/10.1175/BAMS-83-11-1631

[8] S.-C. Pai, C.-C. Yang, J. P. Riley. Formation kinetics of the pink azo dye in the determination of nitrite in natural waters. Anal. Chim. Acta 232 (2) (1990) 345–349. https://doi.org/10.1016/S0003-2670(00)81252-0

[9] H. P. Hansen, F. Koroleff. Chapter 10 Determination of nutrients. In: K. Grasshoff, K. Kremling, M. Ehrhardt (eds.), Methods of seawater analysis (3rd Edition). WILEY−VCH Verlag GmbH (1990). https://doi.org/10.1002/9783527613984.ch10

[10] L.-W. Zheng, W.-D. Zhai. Excess nitrogen in the Bohai and Yellow seas, China: distribution, trends, and source apportionment. Sci. Total Environ. 794 (2021) 148702. https://doi.org/10.1016/j.scitotenv.2021.148702

[11] C. L. Sabine, R. A. Feely, R. M. Key, et al.. Distribution of anthropogenic CO_2 in the Pacific Ocean. Global Biogeochem. Cycles 16 (4) (2002) 1083. https://doi.org/10.1029/2001GB001639

[12] N. Gruber, J. L. Sarmiento, T. F. Stocker. An improved method for detecting anthropogenic CO_2 in the oceans. Global Biogeochem. Cycles 10 (4) (1996) 809–837. https://doi.org/10.1029/96GB01608

[13] L. A. Anderson, J. L. Sarmiento, 1994. Redfield ratios of remineralization determined by nutrient data analysis. Global Biogeochem. Cycles 8 (1) (1994) 65–80. https://doi.org/10.1029/93GB03318

[14] C. Deutsch, N. Gruber, R. M. Key, et al. Denitrification and N_2 fixation in the Pacific Ocean. Global Biogeochem. Cycles 15 (2) (2001) 483–506. https://doi.org/10.1029/2000GB001291

[15] T. Tsubouchi, T. Suga, K. Hanawa, 2016. Comparison study of subtropical mode waters in the world ocean. Front. Mar. Sci. 3 (2016) 270. https://doi.org/10.3389/fmars.2016.00270
